# Supplementary material for: Comparative Evaluation of Four Commercially Available Immunoassays for Therapeutic Drug Monitoring of Infliximab and Adalimumab
Source: Int J Mol Sci. 2023 Jun 20;24(12):10379. doi: 10.3390/ijms241210379 (PMC10299129; doi:10.3390/ijms241210379)
Supplement: Supplementary file 1 [file ijms-24-10379-s001.zip › ijms-2445011-supplementary.pdf]

Supplementary Figure

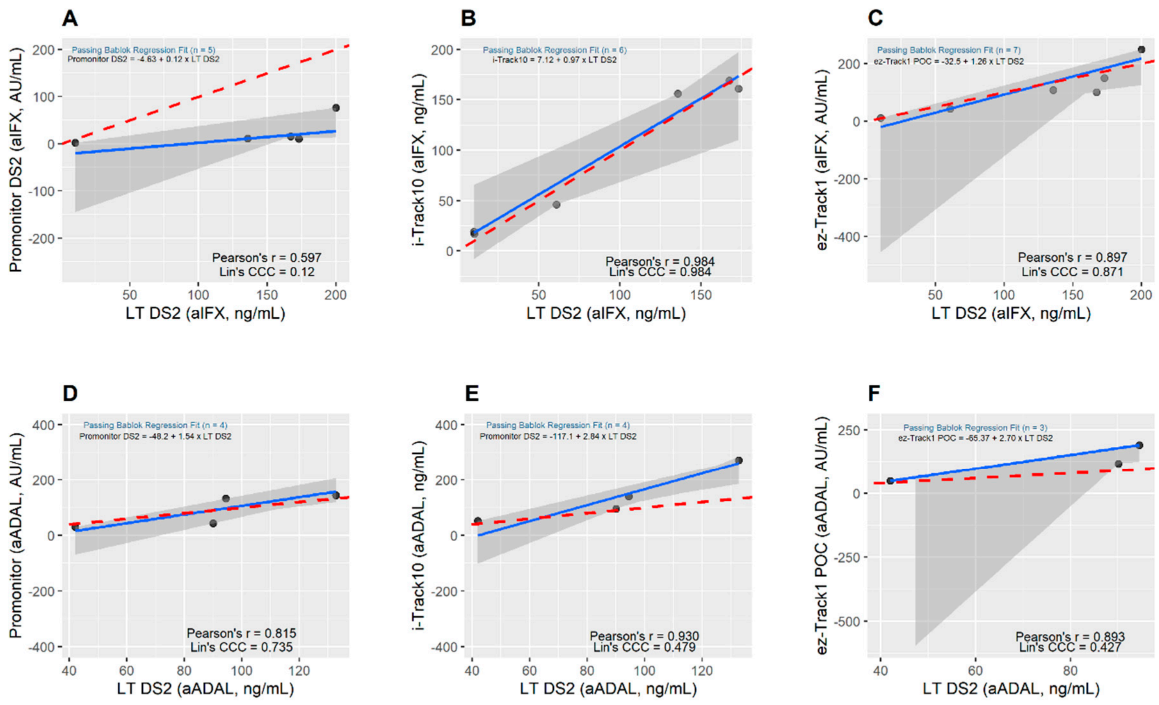

Figure S1. Passing-Bablok regression for aIFX, aADAL.
Passing-Bablok regression fit between LT DS2 and Promonitor DS2/i-TRACK10®/ez-Track1 POC immunoassays for aIFX (**A-C**)**,** aADAL (**D-F**). Pearson's r and Lin's concordance correlation coefficient (CCC) values are shown. The solid blue lines indicate the Passing-Bablok regression. Red dashed lines is identity line: *y* =*x*. Grey shade areas are the 95% confidence bounds calculate with the bootstrap (quantile) method.

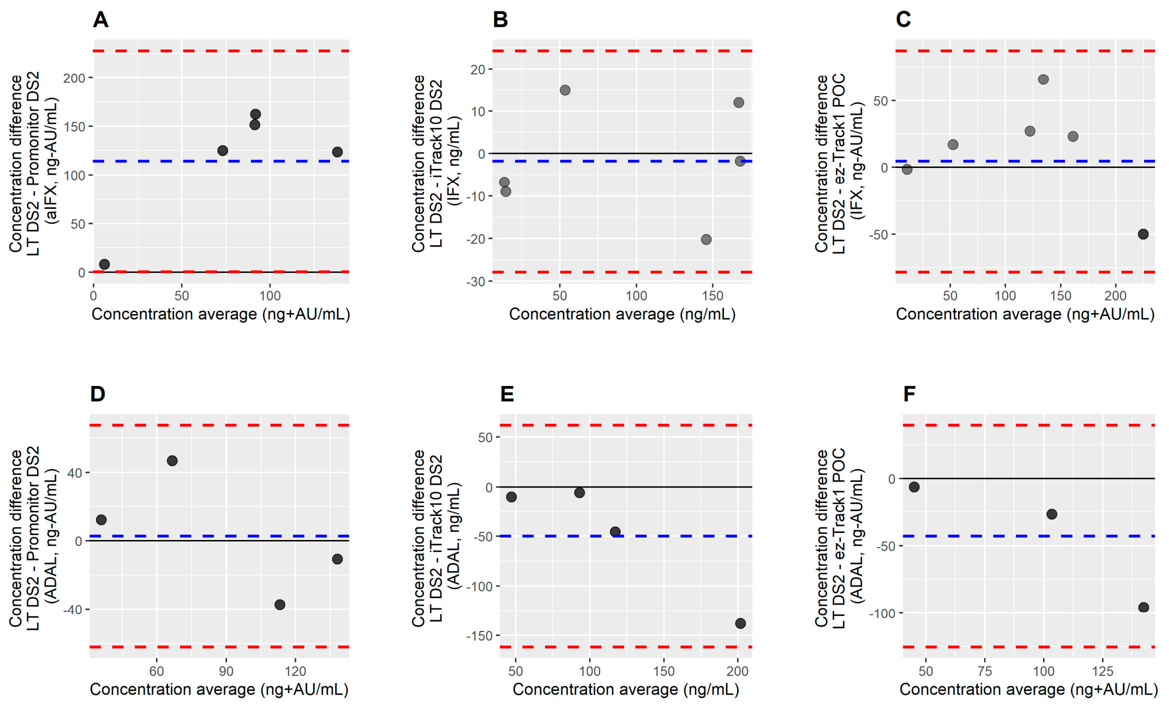

Figure S2. Bland–Altman analysis for aIFX/aADAL measurements

Bland–Altman analysis verifies the difference in aIFX/aADAL measurements between LT DS2 and Promonitor DS2 (**A**/**D**)/i-TRACK10® (**B**/**E**)/ez-Track1 POC (**C**/**F**). The difference between the two measurements is plotted on the y-axis, and the average of the two measurements on the x-axis. Dashed blue lines represent the bias and dashed red lines the 95% LOA for each comparison.
